# Identification of the HSP20 Gene Family in *L. barbarum* and Their Contrasting Response to Heat Stress Between Two Varieties

**DOI:** 10.3390/genes16040440

**Published:** 2025-04-08

**Authors:** Qichen Wu, Yuejie Wang, Zixin Mu

**Affiliations:** 1 College of Life Sciences, Northwest A&F University, Yangling 712100, China; wuqichen2021@163.com; 2 College of Grassland Agriculture, Northwest A&F University, Yangling 712100, China; wangyuejie2019@163.com; 3 College of Life Sciences & Technology, Tarim University, Alar 843300, China; 4 State Key Laboratory Incubation Base for Conservation and Utilization of Bio-Resource in Tarim Basin, Tarim University, Alar 843300, China

**Keywords:** *Lycium barbarum*, heat shock protein, genome-wide, heat stress

## Abstract

**Background**: Small heat shock proteins (*sHsps*), particularly *Hsp20* family members, are pivotal for plant thermotolerance and abiotic stress adaptation. However, their evolutionary dynamics and functional roles in *Lycium barbarum* (goji berry), a commercially significant stress-tolerant crop, remain uncharacterized. This study aims to comprehensively identify *LbHsp20* genes, delineate their evolutionary patterns, and decipher their regulatory mechanisms under heat stress to accelerate molecular breeding of resilient cultivars. **Methods**: Forty-three *LbHsp20* genes were identified from the goji genome using HMMER and BLASTP. Phylogenetic relationships were reconstructed via MEGA-X (maximum likelihood, 1000 bootstraps), while conserved motifs and domains were annotated using MEME Suite and InterProScan. Promoter cis-elements were predicted via PlantCARE. Heat-responsive expression profiles of candidate genes were validated by qRT-PCR in two contrasting lines (N7 and 1402) under 42 °C treatment. **Results**: The *LbHsp20* family clustered into 14 subfamilies, predominantly cytoplasmic (subfamilies I–VII). Chromosomal mapping revealed a tandem duplication hotspot on Chr4 (12 genes) and absence on Chr9, suggesting lineage-specific gene loss. All proteins retained the conserved α-crystallin domain (ACD), with 19 members harboring the ScHsp26-like ACD variant. Promoters were enriched in stress-responsive elements (HSE, ABRE, MYC). Heat stress induced significant upregulation (>15-fold in *LbHsp17.6A* and *LbHsp18.3*) in N7, whereas 1402 showed weaker induction (<5-fold). Subfamily specific divergence was observed, with cytoplasmic subfamily I genes exhibiting the strongest heat responsiveness. **Conclusions**: This study unveils the evolutionary conservation and functional diversification of *LbHsp20* genes in *L. barbarum*. The tandem duplication-driven expansion on Chr4 and subfamily specific expression patterns underpin their roles in thermotolerance. These findings establish a foundation for engineering climate-resilient goji varieties.

## 1. Introduction

Plants face escalating threats from climate-driven heat stress, which disrupts growth and productivity [1]. To mitigate these challenges, *Hsps* particularly the ATP-independent small *Hsp20* family (15–42 kDa), serve as critical molecular chaperones across eukaryotes and prokaryotes [2,3,4,5]. Unlike other *Hsps* (*Hsp100/90/70/60*), *Hsp20* uniquely forms large oligomeric complexes (200–800 kDa) that stabilize denatured proteins under stress [6,7,8,9]. Its conserved α-crystallin domain (ACD)—comprising β-sheets, a hydrophobic β6 loop, and substrate-binding motifs—enables recognition of misfolded proteins, while N/C-terminal variations drive functional diversification [10,11,12,13].

Plant *Hsp20* genes exhibit remarkable lineage-specific expansion, with counts ranging from 19 in Arabidopsis to 51 in soybean, reflecting adaptation to ecological niches [14,15,16,17]. This expansion correlates with structural plasticity: while the ACD is conserved, terminal regions vary, enabling tissue-specific roles (e.g., root stress response, fruit development) [18,19,20]. Notably, crops like tomato (42 *Hsp20s*) and chili pepper (35 *Hsp20s*) show elevated gene numbers compared to model species, suggesting selection for stress resilience in agricultural systems [21,22,23,24].

Despite its agronomic importance, *L. barbarum* (goji berry)—a xerophytic medicinal crop with exceptional drought tolerance—remains underexplored for *Hsp20* biology [25,26,27,28,29,30]. Climate-induced heat stress now threatens its yield and quality, causing fruit abortion, premature ripening, and anthracnose susceptibility [31,32,33,34,35]. However, no studies have characterized its *Hsp20* family or linked gene evolution to desert adaptation [36,37]. Leveraging chromosome-scale genomics, this work identifies *LbHsp20* members, resolves their structural/regulatory innovations, and deciphers their roles in thermotolerance, bridging a critical gap between evolutionary genomics and stress-resilient crop breeding [38,39].

Currently, there is insufficient research focused on identifying the *Hsp20* gene family in *L. barbarum* and examining its response to heat stress. Using new whole genome sequencing technology, this study was the first to deeply analyze the *Hsp20* gene family in *L. barbarum* from Ningxia. By combining genomic data and bioinformatics, researchers identified family members and explained their sequence features, chromosome locations, and evolutionary relationships. They also looked at gene activity in different tissues and how these genes respond to environmental stresses, especially heat stress. The study not only figured out the order of *LbHsp20* but also how different species are related to each other. This study created a map of how *Hsp20* works at the molecular level and provided a basis for further exploring how this group of genes functions and can be used in breeding plants that can better handle stress, enabling heat-resilient crop breeding in arid regions.

## 2. Materials and Methods

### 2.1. Plant Material and Heat Stress Treatment

*L. barbarum* (China Virtual Herbarium Collection Barcode, PE 02040469) (1402, N7) was taken from a collection of plants at the Academy of Agricultural and Forestry Sciences in Ningxia Hui Autonomous Region, China, located at 38°08′ N, 106°09′ E, 1100 m above sea level. Different parts of the *L. barbarum* plant, such as roots, stems, leaves, flowers, and fruits, were gathered. Plants with identical genes that were grown in a greenhouse for about 5 weeks were moved to a special growth room. After one week of growing in a controlled setting (at 25 °C), the young plants were split into two groups. The study had two groups: one group was kept in normal conditions, while the other group was moved to a hot environment at 42 °C to see how heat stress affected them. Leaf samples from the seedlings were taken from the treatment groups at five different times: 0, 1, 3, 6, 12, and 24 h. Three separate tests were performed for each time point. All the samples were quickly frozen using liquid nitrogen and kept in a very cold fridge at −80 °C for later RNA extraction and analysis.

### 2.2. Genome-Wide Characterization of the LbHsp20 Gene in L. barbarum

**Figure 1:**
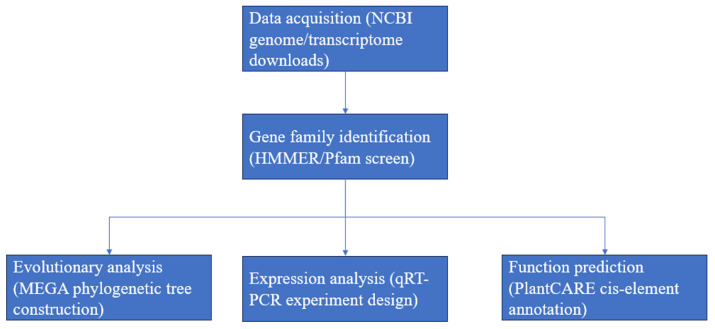
Research technical route. From genomic data acquisition (NCBI) → HMMER gene screening → MEGA evolutionary analysis → PlantCARE promoter prediction → qRT-PCR validation. Arrows are bioinformatics steps, and blue arrows in the image are experimental validation.

The whole *L. barbarum* protein grouping was downloaded from NCBI (https://www.ncbi.nlm.nih.gov/datasets/taxonomy/112863/, accessed on 5 February 2024) (Figure 1). Candidates for *LbHsp20* were found by using a method called Hidden Markov Model (HMM) analysis. We downloaded the HMM profile protein family database for Hsp20 (PF00011) from Pfam (http://pfam-legacy.xfam.org, accessed on 23 February 2024) and used it to search for protein sequences in *L. barbarum* with a significance level of *p* < 0.001. After getting rid of all unnecessary sequences, the potential *Hsp20* protein was sent to CDD, Pfam, and SMART for analysis to confirm that it kept the *Hsp20* structure. We removed sequences with predicted proteins that do not have the *Hsp20* structural domain or that weigh less than 15 kDa or more than 42 kDa. All non-repetitive and high-confidence genes were assigned to *LbHsp20s*. The *LbHsp20* genes were named based on where they are located on the pseudomolecule.

### 2.3. Analysis of the Conserved Motifs of the LbHsp20 Family in L. barbarum

In this study, we used a detailed analysis approach to look at the *Hsp20* gene family in *L. barbarum*. First, we calculated key details about the proteins, like the number of amino acids, molecular weight, isoelectric point, and instability index, using the ExPASy ProtParam tool. We considered proteins with an instability index over 40 to be unstable. Then, we checked the structure of the α-crystallin proteins by validating their structural parts with the Pfam and SMART databases to confirm that these functional areas were biologically valid. The MEME Suite (version 4.0) was used to find similar patterns in sequences. We set it to look for up to 10 patterns, with each pattern being between 6 and 200 amino acids long. We also used the InterProScan platform to check what these patterns might do across different species. This process helped us understand how the patterns are related to chromosomes in different species. We labelled the data, and then created a reliable list of *LbHsp20* genes that includes information about where they are located on the chromosomes. This combined analysis approach greatly enhanced the accuracy of identifying gene families and made functional predictions more reliable by checking data from different sources.

### 2.4. Phylogenetic Analysis and Classification of the LbHsp20 Gene in L. barbarum

The evolutionary characteristics of the *LbHsp20* gene were studied by comparing different species. Researchers gathered the complete *Hsp20* sequences from Arabidopsis, tomato, chili pepper, and rice, and combined them with the *LbHsp20* data to create a dataset. They then used this dataset to build a phylogenetic tree using a method called maximum likelihood, with the help of software called ClustalW2 (v2.1) for comparing the sequences. Using the rules of topological conservatism and the classification criteria for *Hsp20* species, we divided the *LbHsp20* subfamily. We created a clearer version of the phylogenetic tree using the ChiPlot website (https://www.chiplot.online, accessed on 7 February 2024). The analysis method was checked by comparing similar structures across different species to make sure the classification system is reliable.

### 2.5. Chromosome Localization and Gene Duplication

We retrieved genome annotation files from NCBI’s *L. barbarum* genome database and used TBtools (v2.057) software to summarize their physical locations into a chart showing the number and location of chromosomes for each sequence in the genome. The covariance analysis of the *L. barbarum* genome was performed using MCScanX (v1.0), and the *Hsp20* gene sequence was first extracted by gene annotation. Then, homologous gene pairs were constructed by BLASTP against other genes in the genome, and the covariance relationship was visualized using MCScanX software.

### 2.6. RNA Isolation and Expression Analysis of the LbHsp20 Gene in L. barbarum

Total RNA was extracted from different tissue samples using TRIzol reagent provided by Invitrogen. Then, we checked how pure the RNA was using a NanoDrop 2000 spectrophotometer for the experiments that followed. A total of 1 μg of RNA was converted into cDNA using the FastKing RT kit (TIANGEN, Beijing, China). Real-time fluorescence measurements were performed using the CFX96 Touch system (BIO-RAD, Hercules, CA, USA). The reaction used 0.1 micrograms of cDNA and SuperReal PreMix (SYBR Green, TIANGEN, Beijing, China). The temperature settings for the process were as follows: first, heat to 95 °C for 15 min, then do 40 cycles of heating to 95 °C for 10 s, cooling to 60 °C for 30 s, and warming to 72 °C for 32 s. LbACTIN1 was used as a standard reference gene, and the relative expression of the target gene was measured using the 2^−ΔΔCT^ method. The experiment had three separate biological samples and two sets of tests for each sample. We used a melting curve analysis to check that the results were accurate and could be repeated.

### 2.7. Search for Cis-Acting Elements in the Promoter of the LbHsp20 Gene of L. barbarum

This research focused on specific regions of DNA situated 1000 base pairs upstream from the initiation points of various genes, which play a role in gene regulation. We found important regulatory features, like those that respond to light, hormones, and stress, using the PlantCARE database. We created a distribution matrix of regulatory elements to measure how many there were and where each type of functional element (like HSE, ABRE, MYB, etc.) was located. This helped us make a digital signature that offers information for studying the network that controls gene expression.

## 3. Results

### 3.1. Identification and Analysis of LbHsp20 Gene Family Members in L. barbarum

Utilizing the Hidden Markov Model (HMM) analysis on the gene database of *L. barbarum*, we discovered 45 gene sequences related to *Hsp20*. After further validation with the Pfam and SMART databases, sequences that did not contain the typical α-crystallin structural domain (ACD) and whose molecular weights were outside the range of 15–42 kDa were excluded, and 43 *L. barbarum Hsp20* genes were finally confirmed (Table 1). These genes were named *LbHsp20* genes, and their detailed information, including gene name, gene ID, chromosomal location, open reading frame (ORF) length, number of amino acids, molecular weight, isoelectric point (pI), and instability index, are listed in Table 1. The amino acid length of the *LbHsp20* proteins ranged from 137 amino acids (*LbHsp15.8*) to 243 amino acids (*LbHsp27.6*), and molecular weights ranged from 15.8 kDa (LbHsp15.8) to 27.62 kDa (*LbHsp27.6*). These genes are distributed on 12 chromosomes of *L. barbarum*. The predicted isoelectric point (pI) of the *LbHsp20* protein ranged from 4.61 (*LbHsp24.2*) to 10.46 (*LbHsp22.9*). Twelve of the *LbHsp20* proteins were considered stable based on the instability index prediction, and the remaining proteins were classified as unstable.

### 3.2. Gene Structure of the LbHsp20 Gene

**Figure 2:**
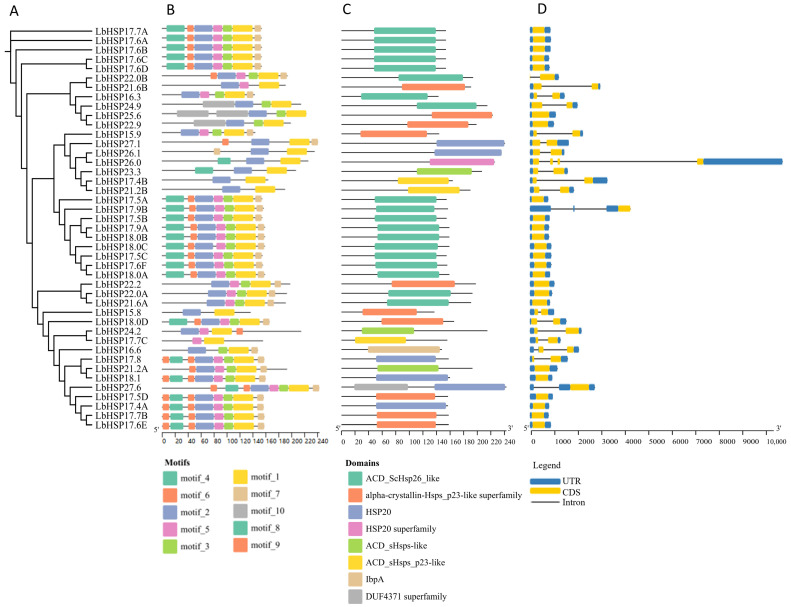
In this study, we reveal the *LbHsp20* protein family properties through multidimensional molecular evolutionary feature resolution: (**A**) the reconstructed phylogenetic topology based on the MEGAX platform (Bootstrap = 1000), which shows the divergence time sequences of different subfamilies; (**B**) the MEME algorithm identifies 10 characteristic motifs (Motif1–10), whose spatial distributions of the color-block markers reflect the linear arrangement pattern of functional blocks, and the calibration ruler precisely indicates the range of length variation in each isoform; (**C**) in the structural domain annotation map, gray tracks characterize the full-length sequences, and colored modules correspond to the conserved functional units such as α-crystallin; (**D**) the exon–intron architecture resolved by GSDS 2.0 reveals that the x-axis quantitatively characterizes the length difference in splice units (50–2500 bp), which reveals the gene structure evolutionary trajectories. All the analysis modules were visualized and integrated by GSDS 2.0.

In this study, the evolutionary relationships of 43 *LbHsp20* genes were analyzed by multiple sequence comparison, and a maximum likelihood tree was constructed based on the amino acid sequences (Figure 2A). To further resolve the structural features of the *LbHsps* proteins, the MEME (5.5.4) software was used to perform motif analysis of the 43 *LbHsps*, and 10 conserved motifs were identified, whose lengths ranged from 8 to 113 amino acids (Figure 2B). As shown in Table 2, the details of 10 of these defined motifs have been systematically characterized. By integrating the analysis results from Pfam, CDD, and SMART databases, all *LbHsp20* proteins were found to contain a combination of motif 1 and motif 2 features. Notably, 19 of the 43 *LbHsp20* members have the conserved ACD_ScHsp26_like structural domain (Figure 2C).

Comparative analysis of genomic DNA and *LbHsp20* full-length cDNA revealed the structural features of introns/exons and their phase information. The results showed that 55.8% (24) of the *LbHsp20* genes contained no introns, 37.2% (16) contained one intron, while 6.9% (3) of the genes (including *LbHsp26.0*, *LbHsp17.5A* and *LbHsp16.6*) contained two or more introns (Figure 2D). Of these, *LbHsp26.0* contains three introns, *LbHsp17.5A* and *LbHsp16.6* each contain two introns.

It has been shown that certain important parts of the ACD structure might help in making larger groups of proteins that play a key role in controlling the activity of the *Hsp20* gene, which acts as a chaperone. In this study, we found that the C-terminal region of the *Hsp20* protein of *L. barbarum* contained a highly conserved amino acid sequence (KKPEVKAIDIS), suggesting that this region may play a key role in the stability and functional regulation of the protein. In addition, the conservativeness analysis showed that the N-terminal region (FMRRFRLPENAKMDAIKAAMENGVLT) of the *Hsp20* protein of *L. lycii* was highly conserved among the homologous sequences of different plants. It was further verified by the ConSurf tool that this region is highly conserved and may be closely related to the function of small heat stress proteins and their regulatory mechanisms in stress response. These findings provide new insights for a deeper understanding of the structure and function of the *Hsp20* protein (Table 2).

### 3.3. Phylogenetic Analysis of the LbHsp20 Family

**Figure 3:**
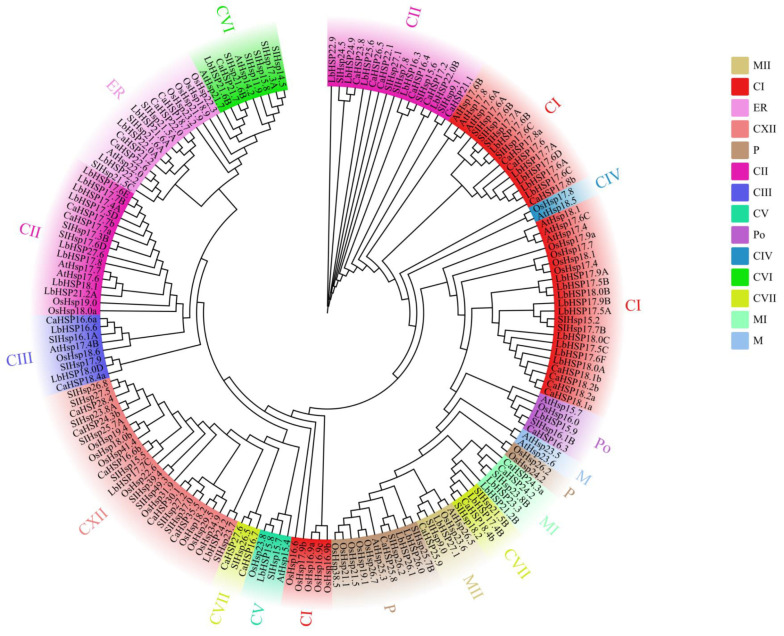
Phylogenetic analysis of 43 *L. barbarum Hsp20* (*LbHsp20*) homologs with Capsicum annuum, rice, Arabidopsis thaliana, and tomato.

To investigate the evolutionary relationships of the *Hsp20* gene family in *L. barbarum*, Capsicum annuum, Solanum lycopersicum, Arabidopsis thaliana, and Oryza sativa, we performed multiple sequence comparisons of the amino acid sequences of 43 *Hsp20* proteins in *L. barbarum*, 35 *Hsp20* proteins in Capsicum annuum, 42 *Hsp20* proteins in Solanum lycopersicum, 19 Hsp20 proteins in Arabidopsis, and 35 *Hsp20* proteins in rice, and constructed a rootless phylogenetic tree. A total of 19 and 35 *Hsp20* proteins in rice were subjected to multiple sequence comparisons of amino acid sequences, and a rootless phylogenetic tree was constructed (Figure 3). Based on the phylogenetic analysis, 174 *Hsp20* proteins were classified into 14 different subfamilies, including 42 cytoplasmic type I (CI), 38 cytoplasmic type II (CII), 8 cytoplasmic type III (CIII), 2 cytoplasmic type IV (CIV), 4 cytoplasmic type V (CV), 9 cytoplasmic type VI (CVI), 7 cytoplasmic type VII (CVII), 2 mitochondrial types (M), 5 mitochondrial I (MI), 5 mitochondrial II (MII), 13 plastid (P), 5 peroxisomal (Po), and 15 endoplasmic reticulum (ER). Phylogenetic analyses showed that most *Hsp20* members (including 29 *StHsp20s*) were classified into the CI-CVII subfamily, suggesting that cytoplasmic lysosomes may be the main functional compartment of plant *Hsp20*. Notably, *LbHsp20* members were more closely related to members of the same subfamily in different species than they were to other *Hsp20* members within the same species, a phenomenon that suggests a high degree of covariance between members of the same *Hsp20* subfamily across species and implies that these genes may have undergone conserved functional divergence during evolution.

### 3.4. Chromosomal Localization and Gene Duplication

**Figure 4:**
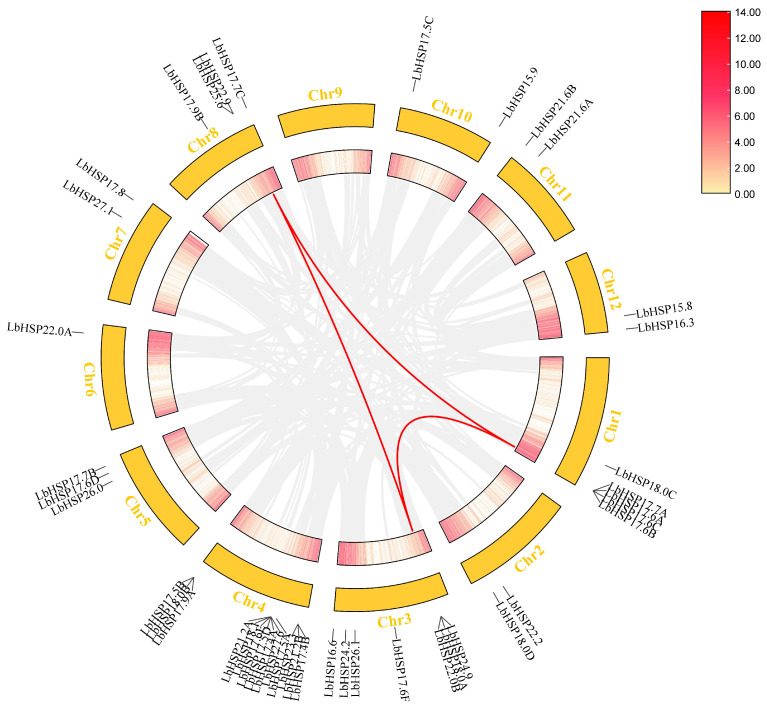
Chromosomal localization and covariance analysis of the *LbHsp20* gene. Chromosome distribution map: distribution of *Hsp20* genes on LBP chromosomes, numbers indicate gene positions; intragenomic covariance: red lines connecting *Hsp20* gene pairs with covariance, highlighted; red lines in the boxes represent gene densities.

The 43 *LbHsp20* genes were distributed on 11 *L. barbarum* chromosomes, and chromosome 9 was not distributed (Figure 4). Among these 43 *LbHsp20* genes, chromosome 4 was the most densely distributed, containing 13 LbHsp20 genes, accounting for 30.23% of all genes, indicating that this chromosome may be the main region for the expansion of the *LbHsp20* gene family. Chromosome 3 was distributed with 7 *LbHsp20* genes, accounting for 16.28% of the total number of genes, showing a higher gene density. Chromosomes 1, 8 and chromosomes 5, 4, and 3 *LbHsp20* genes were distributed on chromosomes 1, 8, and 5, accounting for 11.63%, 9.30%, and 6.98% of the total, respectively. Chromosomes 2, 7, 10, 11, and 12 each had two *LbHsp20* genes per chromosome, accounting for 4.65% of the total number of genes, and chromosome 6 had only one *LbHsp20* gene, accounting for 2.33% of the total number of genes, while chromosome 9 did not have any detectable distribution of *LbHsp20* genes, a phenomenon that may be related to chromosome-specific structural variants, functional partitioning, or the presence of the *LbHsp20* gene. Multiple pairs of transchromosomal *Hsp20* covariates were detected within the *L. barbarum* genome, including *LbHSP17.7A* and *LbHSP24.9*, *LbHSP17.6C* and *LbHSP22.0B*, *LbHSP17.6A* and *LbHSP22.9*, *LbHSP17.7A* and *LbHSP25.6*, and *LbHSP24.9* and *LbHSP25.6*, suggesting expansion through genome duplication and selection by purification.

### 3.5. Analysis of Cis-Elements in the LbHsp20 Promoter

**Figure 5:**
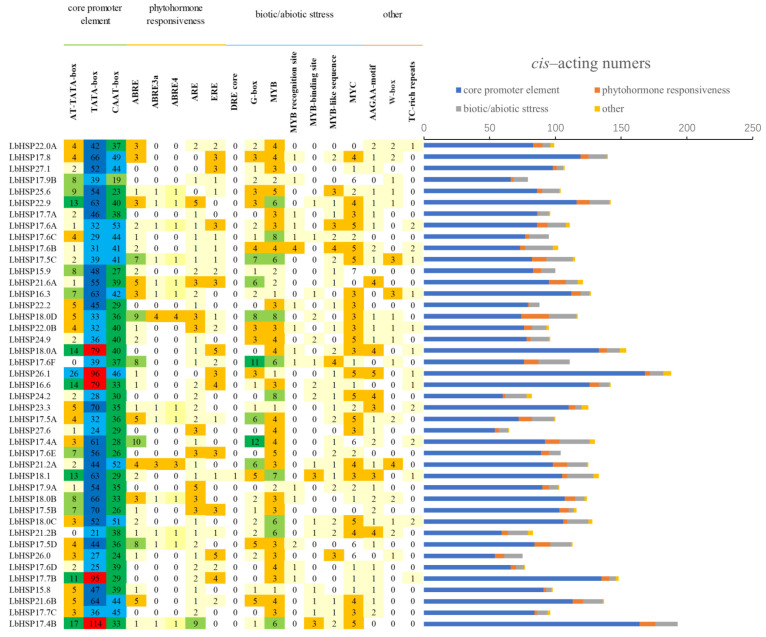
Analysis of the promoter region of the *LbHsp20* gene reveals three types of core regulatory modules: hormone response, response to adversity, and photoperiodic regulatory elements.

Promoter cis-acting elements, as key binding regions of transcription initiation factors, play a crucial role in the regulation of gene expression. In order to deeply investigate the potential biological functions of the 43 *LbHsp20* genes, this study systematically predicted the cis-acting elements in the 1 kb promoter region upstream of the corresponding genes using the PlantCARE database (as shown). The analysis results showed that, in addition to the core promoter elements such as TATA-box and CAAT-box, a variety of specific cis-acting elements related to hormone response, adversity stress, and tissue and organ development were identified, which may play important regulatory roles in the activation and induction of *LbHsp20* gene expression (Figure 5, Supplementary Materials).

In terms of phytohormone response elements, including abscisic acid (ABA) response element (ABRE) and ethylene response element (ERE) were identified. Among the biotic/abiotic stress response elements, MYC, MYB, W-box, TC-rich repeats (involved in defense and stress response), the drought osmotic stress-inducing element (DRE core), the anaerobic-induced regulatory element (ARE), and the MYB binding site were identified, which may be closely related to the tolerance and response mechanism of plants to biotic and abiotic stresses. In addition, a light-responsive element (G-box), an oxidative defense pathway-associated element (AAGAA-motif), and a circadian regulator were also identified.

### 3.6. Expression Profile of LbHsp20 Gene Induced by Heat Stresses

**Figure 6:**
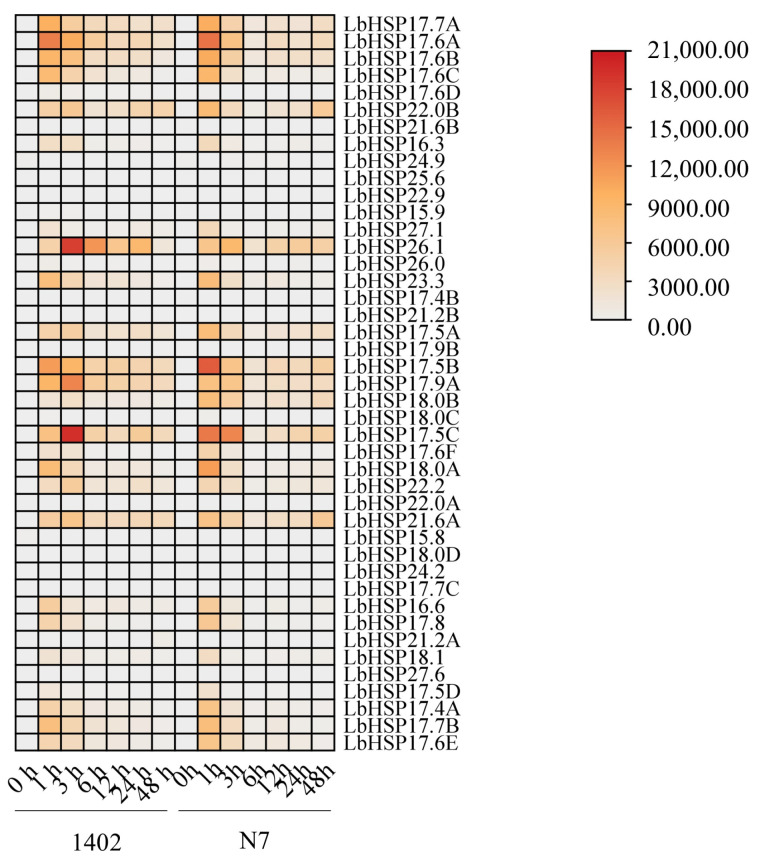
Specific expression of the *LbHsp20* gene. Using a method known as qRT-PCR with specific primers, we evaluated the expression of the *LbHsp20* gene in the leaves of seedlings from the 1402 and N7 varieties. After performing qRT-PCR on three separate occasions, we assessed the expression alterations using the 2^−ΔΔCT^ method, with the LbACTIN1 gene serving as a reference control. The values are the results from three separate tests.

To better understand how the *LbHsp20* gene family is controlled under high temperatures, we measured the levels of 43 *LbHsp20* genes in the leaves of two types of plants (N7 and 1402) using a method called qRT-PCR in this study (Figure 6, Supplementary Materials). The experiment included three separate tests using different samples and two repeated tests to make sure the results were reliable and could be repeated. The results showed that the *LbHsp20* gene family in both lines exhibited significant heat stress response characteristics under continuous heat stress treatment at 42 °C. Among them, the majority of genes (e.g., *LbHsp17.7A*, *17.6A*, *17.6B*, *17.6C*, *17.6D*, *22.0B*, *17.5B*, *17.9A*, *18.0B*, *18.0C*, *17.5C*, *17.6F*, *18.0A*, *22.2*, *17.5D*, *17.4A*, *17.7B*, and *17.6E*) showed significant response characteristics to heat stress in short-term heat stress (1 h), and this high expression state persisted until the end of the stress treatment. In contrast, *LbHsp24.9*, *22.0A*, and *15.8* showed a down-regulated expression pattern. Notably, genes such as *LbHsp22.9*, *17.9B*, *15.8*, *18.0D*, *24.2*, *17.7C*, and *27.6* did not show significant expression differences before and after heat stress treatment. In addition, comparative analysis showed that the overall expression levels of the two lines of *L. barbarum* were different after heat stress treatment, suggesting that there may be different heat stress response mechanisms between the different lines, providing a basis for comparing their heat tolerance. These findings provide an important experimental basis for further elucidating the molecular mechanism of the *LbHsp20* gene family in plant heat stress responses.

### 3.7. Expression Pattern of LbHsp20 Gene in Different Tissues

**Figure 7:**
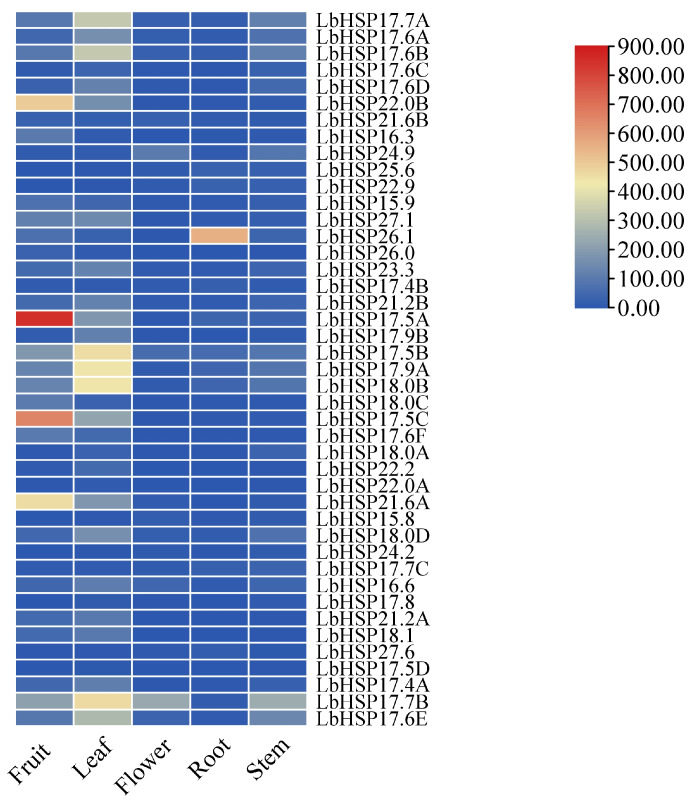
Tissue-specific expression of *LbHsp20* genes. The expression of the *LbHsp20* gene in different tissues of *L. barbarum* was evaluated using qRT-PCR with specific primers.

This research focused on examining the expression of the *LbHsp20* gene family across various plant tissues—roots, stems, leaves, flowers, and fruits—utilizing the qRT-PCR technique (Figure 7). The results showed that the family members showed significant spatial expression heterogeneity: *LbHsp22.0B*, *Hsp17.5A*, *LbHsp17.5C* and *LbHsp21.6A* were specifically highly expressed in fruits, suggesting that they may be involved in the regulation of fruit development or ripening; LbHsp26.1 had a significant expression dominance in the roots, while *LbHsp17.5B* had the highest transcriptional had the highest abundance. Notably, although *LbHsp17.7B*, *LbHsp17.5B*, and *LbHsp24.9* showed localized expression characteristics in flowers, most of the family members (e.g., *LbHsp17.4A* and *LbHsp22.0B*) were expressed at significantly lower levels in this tissue than in other tissues. Of particular importance, *LbHsp17.5B* maintained a constitutively high expression pattern in all tissues tested, suggesting that it may play a central role in the maintenance of basal cellular homeostasis. These findings reveal the functional differentiation of this *Hsp* gene family in different tissues of *LbHsp* and provide an important basis for resolving its molecular regulatory network.

## 4. Discussion

This study systematically analyzed the 43 *LbHsp20* genes in *L. barbarum*, revealing the evolutionary, structural, expression regulation, and functional diversity of this gene family. Below, we discuss the evolutionary relationships, structural features, expression regulation mechanisms, and functional differentiation of *LbHsp20*.

### 4.1. Evolution and Classification of the LbHsp20 Gene Family

Phylogenetic classification of *LbHsp20s* into 14 subfamilies aligns with patterns observed in tomato, rice, and Arabidopsis, where cytoplasmic subfamilies (I–VII) dominate, underscoring the functional conservation of *Hsp20s* in stress adaptation across angiosperms. However, unlike the uniform chromosomal distribution reported in maize, *LbHsp20s* clustered densely on chromosome 4, mirroring expansion hotspots in pepper, suggesting shared evolutionary pressures in Solanaceae. The absence of *LbHsp20s* on chromosome 9 parallels findings in wheat, where gene loss or chromosomal rearrangements likely drove family diversification. Notably, *LbHsp20s* showed stronger homology to orthologs in divergent species than to paralogs within *L. barbarum*, a phenomenon also observed in poplar, indicating lineage-specific neofunctionalization post-speciation [12].

### 4.2. Structural Characterization of the LbHsp20 Protein

All *LbHsp20s* retained motif 1 and motif 2, consistent with the conserved α-crystallin domain (ACD) architecture critical for chaperone activity in Arabidopsis. The ACD_ScHsp26_like domain in 19 members resembles the yeast Hsp26 chaperone system, reinforcing its role in stress-induced protein refolding. The conserved C-terminal KKPEVKAIDIS motif, analogous to the IXI/V motif in rice *Hsp20s*, likely stabilizes substrate binding during thermal stress, while the N-terminal FMRRFRFRLPENAKMDAIKAAMENGVLT sequence shares similarity with ATP-independent chaperone motifs in Glycine max, suggesting evolutionary convergence in maintaining proteostasis under abiotic stress [18].

The *Hsp20* gene family is both conserved and species-differentiated in plants. Conservatism is reflected in the widespread presence of the core α-crystallin structural domain (ACD) and HSE/ABRE promoter elements that maintain basal stress functions. Differences include gene number (19 in Arabidopsis and 43 in *L. barbarum*), chromosomal distribution (evenly distributed in maize and enriched in *L. barbarum* chr4), and functional differentiation (rice flooding tolerance subfamily, LB fruit highly expressed genes). Local replication and regulatory remodeling in Solanaceae drive adaptive evolution, and future integration of mosses and other taxa is needed to resolve early origins.

### 4.3. Mechanisms Regulating the Expression of the LbHsp20 Gene

The enrichment of MYC, ABRE, and ARE cis-elements in *LbHsp20* promotes parallel findings in Arabidopsis and cotton, where these motifs mediate ABA-dependent drought and heat responses. The G-box, implicated in light signaling in tomato, suggests *LbHsp20s* integrate photoperiod cues with stress adaptation, a mechanism less pronounced in maize *Hsp20s*. These results align with studies highlighting genetic background as a key determinant of *Hsp20* expression plasticity [33].

### 4.4. Functional Differentiation of the LbHsp20 Gene

The expression patterns of *LbHsp20* genes under heat stress showed significant functional differentiation. Most genes were upregulated immediately after short-term heat stress, while a few were downregulated or unchanged, indicating different roles in the stress response. Upregulated genes may be involved in protein folding, stability, and complex formation, while downregulated genes could participate in alternative stress pathways or negative regulatory mechanisms. The expression differences among lines suggest that genetic background influences v gene regulation, providing potential targets for genetic improvement to enhance plant stress tolerance [32].

This study also highlighted the dynamic tissue-specific expression patterns of the *LbHsp* gene family, reflecting their functional diversity. For instance, fruit-specific high expression of genes like *LbHsp22.0B* and *Hsp17.5A* may be involved in stress responses during fruit development, while constitutive expression of *LbHsp17.5B* suggests its role in maintaining basal cellular functions. Additionally, the significant expression of *LbHsp26.1* in roots may be related to its function in root stress responses.

## 5. Conclusions

In this study, we looked at all genes in the Lycium *L. barbarum Hsp20* family and identified 43 *LbHsp20* genes. Next, the researchers used bioinformatics and qRT-PCR techniques in the study of the structure of the *LbHsp20* genes, their evolutionary history, chromosomal location, stress-related elements, and how they respond to abiotic stress. Most *LbHsp20* genes are highly responsive to heat stress, and their activity increases rapidly. This means the *LbHsp20* genes are important for helping goji berries withstand high temperatures. A detailed study of the *LbHsp20* gene family in medlar was conducted to understand the role of the *LbHsp20* gene in medlar.

## Figures and Tables

**Table 1:** The list of LbHsp20 members identified.

Gene Name	Version	cds (bp)	Protein Length	Molecular Weight (kD)	Instability Index	Isoelectric Point
LbHsp17.6A	XM_060319191.1	465	154	17.62	43.95	6.56
LbHsp17.7A	XM_060319183.1	465	154	17.65	43.77	6.56
LbHsp17.6B	XM_060319222.1	465	154	17.61	45.55	6.56
LbHsp17.6C	XM_060319213.1	465	154	17.61	52.45	5.89
LbHsp17.6D	XM_060358525.1	465	154	17.58	51.65	5.89
LbHsp17.9A	XM_060354035.1	480	159	17.94	52.55	5.88
LbHsp17.5A	XM_060352452.1	468	155	17.54	57.04	6.56
LbHsp18.0A	XM_060346165.1	480	159	17.97	62.07	7.7
LbHsp17.5B	XM_060354037.1	467	155	17.48	58.55	5.88
LbHsp21.6A	XM_060333068.1	576	191	21.61	42.93	7.9
LbHsp18.0B	XM_060354036.1	480	159	17.96	61.95	5.88
LbHsp17.5C	XM_060330013.1	468	155	17.45	63.75	5.88
LbHsp22.0A	XM_060313235.1	582	193	21.98	43.8	6.54
LbHsp17.8	XM_060315404.1	477	158	17.77	37.34	6.8
LbHsp17.6E	XM_060352575.1	477	158	17.63	35.92	6.55
LbHsp17.7B	XM_060358882.1	477	158	17.68	38.22	7.6
LbHsp17.6F	XM_060347156.1	471	156	17.61	59.4	10.32
LbHsp17.9B	XM_060318377.1	474	157	17.89	62.72	5.42
LbHsp17.5D	XM_060355297.1	474	157	17.5	36.99	7.51
LbHsp18.1	XM_060352578.1	483	160	18.1	29.11	8.43
LbHsp22.2	XM_060342004.1	597	198	22.24	42.4	5.06
LbHsp17.4A	XM_060352574.1	474	157	17.44	35.03	6.54
LbHsp18.0C	XM_060355099.1	480	159	18.04	63.71	5.9
LbHsp27.1	XM_060316795.1	726	241	27.1	56.94	8.43
LbHsp26.1	XM_060347996.1	711	236	26.15	40.27	8.23
LbHsp22.0B	XM_060345998.1	585	194	22.07	37.91	9.52
LbHsp18.0D	XM_060342758.1	501	166	18.09	64.23	5.66
LbHsp21.2A	XM_060352576.1	582	193	21.24	31.51	4.9
LbHsp16.3	XM_060337959.1	432	143	16.26	29.4	7.35
LbHsp27.6	XM_060352573.1	732	243	27.62	34.67	9.28
LbHsp24.9	XM_060346161.1	648	215	24.94	47.9	7.57
LbHsp16.6	XM_060350036.1	447	148	16.63	63.04	5.09
LbHsp22.9	XM_060318505.1	600	199	22.88	51.2	10.46
LbHsp15.9	XM_060330065.1	435	144	15.88	45.37	7.84
LbHspP23.3	XM_060351341.1	624	207	23.48	59.54	5.24
LbHsp25.6	XM_060318504.1	672	223	25.55	50.61	9.78
LbHsp26.0	XM_060358140.1	681	226	26.02	53.8	7.29
LbHsp15.8	XM_060336286.1	414	137	15.8	45.17	5.7
LbHsp24.2	XM_060350504.1	648	215	24.22	50.07	4.61
LbHsp21.2B	XM_060355129.1	573	190	21.23	52.44	9.04
LbHsp21.6B	XM_060332688.1	576	191	21.63	34.03	5.11
LbHsp17.7C	XM_060318664.1	471	156	17.69	16.01	6.28
LbHsp17.4B	XM_060351340.1	495	164	17.74	40.42	5.05

**Table 2:** List of the putative motifs of *LbHsp20* proteins.

Motif	Best Possible Match	Width
1	FMRRFRLPENAKMDAIKAAMENGVLTVTVPKE	32
2	DWKETPEAHVFKVDLPGJKKEEVKVZVEE	29
3	KKNDKWHRMERSSGK	15
4	SFFGGRRSNIFDPFSLDVFDPFEGFPFPN	29
5	DRVLQISGERKREEE	15
6	RETSAFANARI	11
7	KKPEVKAIDIS	11
8	LFHTLQHMMDIAGDDSDKPVN	21
9	MDFRLMGIDNP	11
10	PTIKITRQSQPNRGRQDPYDPSREFYLZNPRSLIAPALSFPQEPPSQAPI	50

## Data Availability

Data are contained within the article.

## Supplementary Materials

The following supporting information can be downloaded at: https://www.mdpi.com/article/10.3390/genes16040440/s1, Table S1: The primer sequences in qRT-PCR; Table S2: The sequences of *cis*-acting element; Table S3: Expression profile of *LbHsp20* gene induced by heat stresses; Table S4: Expression pattern of *LbHsp20* gene in different tissues; Table S5: *LbHsp20* cds.
